# Prevalence of Incontinence in the Black Women’s Health Study

**DOI:** 10.1007/s00192-025-06461-y

**Published:** 2026-01-23

**Authors:** Ukpebo Omosigho, Zhiguo Zhao, Charles Dupont, Rony Adam, Yvette Cozier, Ayush Giri

**Affiliations:** 1 Division of Female Pelvic Medicine and Reconstructive Surgery, Department of Obstetrics and Gynecology, Vanderbilt University Medical Center, Nashville, TN, USA; 2 Department of Biostatistics, Vanderbilt University Medical Center, Nashville, TN, USA; 3 Division of Quantitative and Clinical Sciences, Department of Obstetrics and Gynecology, Vanderbilt University Medical Center, Nashville, TN, USA; 4 Division of Epidemiology, Department of Medicine, Vanderbilt University Medical Center, Nashville, TN, USA; 5 Slone Epidemiology Center, Boston University, Boston, MA, USA; 6 Department of Epidemiology, Boston University School of Public Health, Boston, MA, USA

**Keywords:** Dual Incontinence, Fecal Incontinence, Urinary Incontinence

## Abstract

**Introduction and Hypothesis:**

Several epidemiological studies estimate the prevalence of incontinence, urinary (UI), fecal (FI), and dual (DI) in US women; however, none has specifically examined the prevalence of UI, FI, and DI in a large cohort of self-identified Black women. The primary objective was to estimate the prevalence of the incontinence types in the Black Women’s Health Study (BWHS) and extrapolate these findings to Black women in the USA by integrating BWHS prevalence with the 2020 US Census data.

**Methods:**

Among BWHS participants responding to the biennial follow-up questionnaire administered in 2011, we assessed the prevalence of UI in all women (*N* = 38,100) and the prevalence of FI and DI in post-menopausal women (*N* = 19,244). We then standardized age-specific prevalences from BWHS using the 2020 US Census weights to extrapolate findings to self-identified Black women in the USA. All analyses were conducted using R version 4.3.

**Results:**

Approximately one in two participants reported having UI at least once a year (50.6%). Among post-menopausal women, 14% reported any FI, and 10% reported DI. Extrapolating estimates from BWHS data to the 2020 US Census data for US Black women aged 35 years or older, approximately 5.6 million Black women (95% CI = 5.42–5.80 million) had UI, 784,529 women had FI (95% CI = 0.70–0.91 million), and 608,710 women had DI (95% CI = 0.53–0.72 million).

**Conclusions:**

In this large study among community dwelling Black women, prevalence estimates of UI, FI, and DI are higher than previously reported, translating to over five million affected US Black women.

## Introduction

The prevalence of incontinence, urinary (UI), fecal (FI), and dual incontinence (DI) are poorly estimated in women who identify as Black in the USA. The prevalence of UI is reported at over 50% amongst all women in the USA in recent population based studies [1]. The prevalence of UI for Black women in the USA is more variable in the literature, often reporting a decreased prevalence of UI compared with the general population and other ethnic groups. A National Health and Nutrition Examination Survey (NHANES) study published in 2006 reported Black women as having half the prevalence of UI of white women (20% vs 41%) [2]. Similarly, in 2008, a study of UI among women in Michigan reported the prevalence in Black women as 14.6% compared with 33.1% in white women [3].

Estimates of the prevalence of FI and DI also vary in the reported literature amongst Black women [4–9]. The overall prevalence of FI in the USA is estimated to be approximately 10% in women, with prevalence increasing with age [10]. The prevalence of FI amongst Black women was reported to be approximately 2.9% in the Nurses’ Health Study (NHS); in contrast, the prevalence was 4.3% in white women [6]. The prevalence of DI amongst Black women in this same study was reported to be 3.1% vs 7.3 in white women. Additionally, an NHANES study examining PFDs in people over the age of 50 found that 6% of all women had DI, and estimated that women from ethnic minorities (including non-Hispanic Black, Hispanic, and others) combined into the “other” category had a prevalence of 4.2% [11]. Black women are historically underrepresented in studies related to incontinence, and are often grouped together in the “other” category (including non-Hispanic Black, Hispanic, and Asians, among others) owing to small sample sizes, as noted by the aforementioned studies. As a result, there is uncertainty regarding the prevalence of UI, and especially FI, as well as DI in this population.

Given the substantial impact of incontinence on quality of life, and the dearth of studies in Black women that comprehensively evaluate all types of incontinence (UI, FI, and DI) in this population, it is important to obtain estimates of these prevalences in a sample large enough to provide such estimates. Thus, the primary objective was to determine the prevalence of UI, FI, and DI in the Black Women’s Health Study (BWHS). To provide greater insight into the public health relevance of incontinence, we aimed to estimate the burden of incontinence among US Black women by applying the age-specific prevalence estimates from the BWHS to the corresponding population in the 2020 US Census data.

## Materials and Methods

The BWHS is a national prospective cohort study of 59,000 self-identified US Black women [12]. Black women from across the USA were invited to participate in this study to help to better understand diseases such as breast cancer that cause disproportionate morbidity and mortality compared with other racial ethnic groups. The BWHS is a convenience sample of Black women in the USA. At baseline enrolment in 1995, participants were 21–69 years of age (median = 38 years), and represented all regions of the USA [13]. Participants provided information on demographic characteristics, lifestyle and socioeconomic factors, and medical conditions, through biennial postal questionnaires. Follow-up, to date, has been successful for over 80% of potential person-years through 13 completed biennial rounds of follow-up (through 2021). The BWHS was approved for the current analysis.

## Characterization and Definition of Urinary/Fecal Incontinence/Dual Incontinence

The data used in this analysis are from the 2011 BWHS questionnaire cycle. In 2011, pre-menopausal and post-menopausal BWHS participants were administered separate questionnaires. Both questionnaires inquired about UI, whereas the post-menopausal questionnaire (v2) additionally inquired about FI. UI was ascertained using the following question: “During the past year, how often have you leaked or lost control of your urine?” Frequency was ascertained by the following response: Never, less than once per month, once per month, 2–3 times per month, about once per week, or almost every day. For analytical purposes, UI was defined as any positive response to this question. Women who responded affirmatively were then asked, “When you lose your urine, how much usually leaks?”, with the following response options: “A few drops,” “Enough to wet your underwear,” “Enough to wet your outer clothing,” “Enough to wet the floor”. Subtypes of UI were further defined by a positive response to the following question: “When you lose urine, what is the usual cause?” Response options were “Coughing, sneezing, laughing or doing physical activity,” indicating stress incontinence (SUI); “A sudden urgent need to go to the bathroom,” indicating urgency incontinence (UUI); “Both of the two prior responses equally,” indicating mixed incontinence (MUI), or “In other circumstances,” indicating other incontinence.

Severity of UI was determined using the Sandvik severity index. The Sandvik index is validated and is calculated by multiplying the reported frequency of UI by the amount of leakage [14]. Frequency of UI was assigned a value from 1 to 4, with a higher number indicating greater frequency, and amount of leakage was assigned a value of 1 for a few drops or 2 for more than a few drops. According to the Sandvik index, a total severity score of 1–2 is classified as mild, a score of 3–4 is classified as moderate, and a score ≥ 6 is classified as severe.

The questionnaire for post-menopausal women additionally included questions about accidental bowel leakage, or FI, specifically “On average, how often in the past year have you experienced any amount of accidental bowel leakage?” This question was asked separately for liquid and solid stool. Possible responses were “Never,” “Less than once per month,” “1–3 times per month,” “About once/week,” “Several times per week,” or “Nearly daily”; FI was defined as any loss of liquid or solid stool in the last year. We defined severity as the reported frequency of any leakage of bowel contents: Mild (1–3 times per month or less), Moderate (about once per week), and severe (several times per week or more). Finally, DI was defined as a positive response to questions about both UI and FI in the last year. We also analyzed a subset of women who reported more frequent DI concurrent with both UI and FI occurring at least once per month.

## Statistical Analysis

Participant demographic and clinical characteristics were summarized and compared according to incontinence status (UI, FI, and DI). For continuous variables, median and interquartile ranges were reported, and compared using Wilcoxon rank sum tests. For categorical variables, frequencies (percentages) were reported and compared using Chi-squared tests. Binomial distribution was used to estimate point prevalence and the 95% confidence intervals (CIs) for overall and age-specific incontinence including overall UI, UI subtypes, FI, and DI. Estimates for FI and DI were limited to post-menopausal women. To provide greater insight into the public health relevance of our study, we extrapolated our findings to self-identified Black women in the USA by applying the age-specific prevalence estimates from the BWHS to the corresponding population in the 2020 US Census data. Age-specific weights from the 2010 and 2020 US Census for the African American women were similar, as they are with the BWHS weights as well. Thus, extrapolation was applied to 2020 US Census. We provide an overall prevalence by combining estimates from all age groups to estimate the total number of Black women over the age of 35 with UI in the USA. As the US Census data did not include menopause information, for FI and DI prevalence estimation, we used women aged 55 and older in the BWHS as a surrogate for menopause. Age 55 was used in this analysis because the US census data are reported in 10-year increments by age. All analyses were conducted using R version 4.3 [15].

## Results

**Fig. 1:**
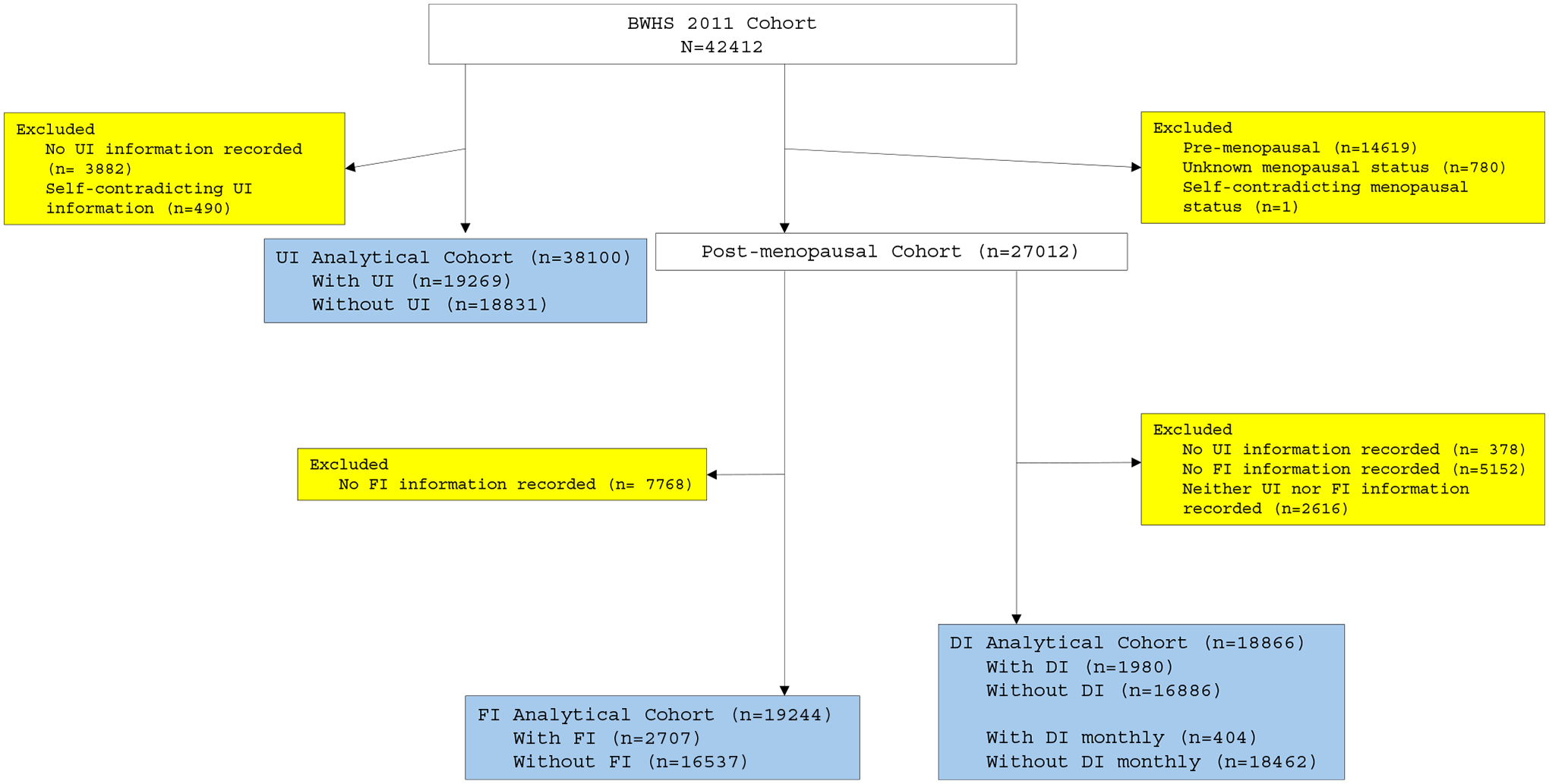
Flow chart. *BWHS* Black Women’s Health Study, *DI* dual incontinence, *FI* fecal incontinence, *UI* urinary incontinence

A total of 42,412 women returned the 2011 BWHS survey (Fig. 1). We excluded 3822 women who did not provide UI data, and 490 women with contradictory UI data (e.g., women who reported “never” leaking urine but report an amount leaked and/or reason for leakage, and/or type of UI). Thus, a total of 38,100 were included in the final analytical sample for UI. The baseline characteristics of women according to incontinence status are included in Table 1. Compared with women without UI, women with UI were slightly older (median age 55 vs 53 years), more likely to be post-menopausal (66% vs 61%), had a higher Body Mass Index (BMI) on average (median 30.7 vs 28.4), were more likely to have type 2 diabetes (T2DM; 21% vs 15%), and depression (26% vs 19%), more likely to use diuretics (44% vs 36%), and more likely to be heavy smokers (median pack-years 10.1 vs 8.5).

The estimated prevalence of any UI was 50.6% (Table 2). The prevalence of any UI increased with age, with approximately 63% of women aged 85 years or older reporting UI. Women aged 35–45 had the lowest prevalence of incontinence at 42%. Among women with UI, 33% reported SUI, 34% UUI, 20% MUI, and 13% reported other incontinence. Most women had mild UI (56.5%), with 24% having moderate UI and 19.5% having severe UI.

A total of 27,012 women completed the post-menopausal (v2) questionnaire (Fig. 1). We excluded 7768 women who did not complete the FI questions, leaving 19,244 post-menopausal women in the FI analytical sample. Finally, the 18,866 post-menopausal women with information on both UI and FI were included in analyses for DI. Post-menopausal women with FI had a slightly higher median BMI (30.6 vs 29.3), were more likely to have T2DM (31% vs 21%), depression (34% vs 22%), hypertension (70% vs 63%), were more likely to use hormone therapy (58% vs 54%), and were more likely to be heavy smokers (13 pack years vs 11 pack years; Table 3). The estimated prevalence of any FI in post-menopausal women was 14%, 39% of whom reported leakage of solid stool and 87% reported leakage of liquid stool. Most women reported mild FI (61%), 20% of whom were classified as having moderate FI and 19% were classified as having severe FI. The prevalence of DI among post-menopausal women was 10.4% (1980 out of 18,866; Table 2). The prevalence of DI in women experiencing symptoms at least once per month for both UI and FI was 2.1% (404 out of 18,866).

The estimated burden of UI, FI, and DI was calculated in Black women in the USA using 2020 Census 5-year estimates (Table 4). By applying the age-specific case rates estimated using BWHS data on the US Census data, we estimate that approximately 5,617,232 (95% CI = 5.42–5.80 million) Black women aged 35 or older currently live with UI (Table 4). Additionally, we estimate that 784,529 women over the age of 55 have FI (95% CI = 0.70–0.91 million) and 608,710 (95% CI = 0.53–0.72 million) women over the age of 55 have DI.

## Discussion

In this cross-sectional analysis of a large cohort of self-identified US Black women aged 35 years or older, we found an overall prevalence of any UI of approximately 50% with the estimated number of Black women experiencing UI symptoms exceeding 5 million. We also found that 14% of post-menopausal women reported experiencing FI and 10.4% reported DI. Contrary to prominent studies that report Black women are less likely to have UI than non-Hispanic white women, we find that UI is just as prevalent among Black women [2, 3, 16, 17]. Our findings regarding the overall prevalence of UI in Black women are similar to those of more recent population-based studies reporting a high prevalence of UI among women using the same frequency definitions [1, 18].

As previously stated, the prevalence of UI in Black women was reported to be 20% in 2006 by an NHANES study, which is half the prevalence reported in US white women [2]. Similarly, a 2008 study of women in Michigan reports the prevalence of UI to be double in white women than in Black women (33.1% vs 14.6%) [3]. Despite having twice the number of Black women as non-Hispanic white women participating in this study, Fenner et al. reported higher rates of incontinence in non-Hispanic white women. Both of the aforementioned studies assess UI yearly in community-dwelling women as in our study [3]. In 2009, Phelan et al. reported the weekly prevalence of incontinence among non-Hispanic white women to be 32% vs 18% in African American women [19]. In contrast, the most recent NHANES study reports UI among non-Hispanic Black women at 54.7% vs 65.6% in non-Hispanic white women [1]. There has been an increase in UI prevalence in both Black and white women, as reported by NHANEs studies; however, the prevalence disparity previously reported is not as profound. The reason for this is poorly understood. Abufaraj et al. reported that the prevalence of SUI from 2005–2018 appears to be stable, with increases in UUI and MUI increasing in women aged 60 and older [20]. Perhaps the increase in prevalence is driven by an increase in UUI amongst Black women over time. Further research may be warranted to understand these previously reported disparities. The trend of UI subtypes in epidemiological studies in the USA report that the most prevalent subtype of UI is SUI followed by UUI and MUI [20]. Similarly, a meta-analysis of UI in women in sub-Saharan Africa found the most prevalent subtype of UI to be SUI followed by MUI and then UUI [21]. In contrast, we found that the most prevalent subtype in our cohort was UUI followed by SUI and MUI. The etiology of Black women in the USA having a higher prevalence of UUI and a lower prevalence of SUI remains poorly understood. A few theories for the observed differences are that Black women have stronger pelvic floor muscles and higher urethral closure pressures than other women [20, 22]. However, why this would apply specifically to US Black women and not to Black women in sub-Saharan Africa, as noted by the trend in primary SUI, is unknown.

The prevalence of FI among community-dwelling individuals in the USA ranges from approximately 7—30% [23, 24]. Our estimated rate of FI at 14% appears to be slightly higher than previously published prevalence estimates in Black women. Berger et al. found a prevalence of 11% among Black women; however, this study included both pre- and post-menopausal women, which may account for the slight difference seen in our study [7]. Similarly, a study of African American women in Michigan reported a prevalence of FI of 6.1% [4]. The women in the aforementioned study had a mean age of 60, which was similar to our population. Our population did include women older than the age of 68, which may account for the increased prevalence. This may represent an increase in prevalence trends over time or community-dependent prevalence, as this prevalence is not nationwide like the BWHS.

The prevalence of DI in US Black women is studied less frequently than either UI or FI alone, with estimates ranging from 1.1% to 5.7% [5, 6, 25]. Thus, our overall prevalence of 10% is one of the highest reported in Black women, to date. We also analyzed a subset of women that reported more frequent DI occurring at least once per month to allow for comparison with other studies. The prevalence of DI in women experiencing symptoms at least monthly for both UI and FI was 2.1%, a finding that is slightly lower than that reported in the Nurses’ Health study (NHS) with the same frequency definition for both urine and stool. DI prevalence was based on approximately 900 Black women compared with over 60,000 white women, highlighting the underrepresentation of Black women in this study. Only 29 Black women met the criteria for FI, and the reported prevalence of DI was 3.1% [6]. Respondents in the NHS were also overall older (62–87 years), compared with our study (36 to 86 years) and offers a potential explanation for the observed difference. Given the impact of DI on quality of life, further research is warranted to understand the true prevalence of DI and the impact specifically in Black women [26].

A major strength of our analysis is the use of a large cohort of self-identified Black women. This cohort is unique as it allows for assessment of UI, FI, and DI within a cohort of women who are often underrepresented in research. Of note, this study is based on self-reported data without clinician diagnosis of UI, FI, and DI, as is the case for many large-scale studies. Although we cannot rule out the possibility that under-reporting of UI may have introduced some degree of bias, the fact that at baseline, 96% of all BWHS participants reported seeing a health care provider within the previous year [27] suggests that any such bias is small. Finally, the BWHS participants are not a random sample of US Black women. The study population underrepresents the 15% of Black women nationally of similar ages who did not complete high school [28]. The participants, however, represent all areas of the USA [13]

In conclusion, this is a large epidemiological study assessing the prevalence of UI, FI, and DI specifically among community-dwelling Black women in the USA. One in two Black women 35 years or older, an estimated 5 million Black women, report UI, which is higher than what is reported by other studies. DI also appears to be higher in US Black women than previously reported.

## Figures and Tables

**Table 1:** Demographic characteristics by urinary incontinence (UI) status

	No UI (*N* = 18,831)	UI (*N* = 19,269)	Total (*N* = 38,100)	*p* value
Age, median (IQR)	53.0 (46.0, 60.0)	55.0 (48.0, 62.0)	54.0 (47.0, 61.0)	< 0.01
BMI, median (IQR)	28.4 (25.0, 33.0)	30.7 (26.6, 35.7)	29.5 (25.8, 34.4)	< 0.01
Obesity, *n* (%)	7338 (40)	10,112 (54)	17,450 (47)	< 0.01
Education, *n* (%)				0.15
< = 12 years	2971 (16)	3164 (16)	6135 (16)	
13–15 years	6584 (35)	6760 (35)	13,344 (35)	
16 + years	9263 (49)	9322 (48)	18,585 (49)	
Health insurance, *n* (%)	16,385 (88)	16,784 (88)	33,169 (88)	0.59
Smoking, *n* (%)				< 0.01
Current	1730 (9)	2005 (10)	3735 (10)	
Past	4456 (24)	5208 (27)	9664 (25)	
Never	12,598 (67)	12,024 (63)	24,622 (65)	
Pack years current smoking, median (IQR)	8.5 (2.8, 17.8)	10.1 (3.5, 19.5)	8.9 (3.1, 18.5)	< 0.01
Hysterectomy, *n* (%)	3153 (17)	3488 (18)	6641 (17)	< 0.01
T2DM, *n* (%)	2810 (15)	4004 (21)	6814 (18)	< 0.01
Parity, *n* (%)				< 0.01
0	4947 (26)	4122 (21)	9069 (24)	
1	5293 (28)	5019 (26)	10,312 (27)	
2	4958 (26)	5445 (28)	10,403 (27)	
3	2263 (12)	2796 (15)	5059 (13)	
4+	1296 (7)	1815 (9)	3111 (8)	
Depression, *n* (%)	3613 (19)	5034 (26)	8647 (23)	< 0.01
Hours per week vigorous exercise, *n* (%)				< 0.01
None	7082 (40)	8176 (46)	15,258 (43)	
< 1–4 h	8535 (49)	8213 (46)	16,748 (47)	
5–10+ hours	1956 (11)	1502 (8)	3458 (10)	
Menopause, *n* (%)				< 0.01
Pre-menopause	7057 (37)	6297 (33)	13,354 (35)	
Post-menopause	11,428 (61)	12,640 (66)	24,068 (63)	
Unknown	346 (2)	332 (2)	677 (2)	
HRT, *n* (%)	6418 (34)	7627 (40)	14,045 (37)	< 0.01
OC use in past 2 years, *n* (%)	1161 (6)	788 (4)	1949 (5)	< 0.01
Diuretic use, *n* (%)	6691 (36)	8491 (44)	15,182 (40)	< 0.01
HTN, *n* (%)	8811 (47)	10,599 (55)	19,410 (51)	< 0.01
HLD, *n* (%)	8340 (44)	9758 (51)	18,098 (48)	< 0.01
History of DVT, *n* (%)	766 (4)	1090 (6)	1856 (5)	< 0.01
History of stroke, *n* (%)	365 (2)	617 (3)	982 (3)	< 0.01

*BMI* Body Mass Index (calculated as weight in kilograms divided by height in meters squared), *Obesity* BMI greater than or equal to 30, *T2DM* type II diabetes mellitus, *HRT* hormone replacement therapy, *OC* oral contraceptive, *HTN* hypertension, *HLD* hyperlipidemia, *DVT* deep venous thrombosis

**Table 2:** Prevalence of incontinence type in the Black Women’s Health Study and breakdown by severity

Type of incontinence, *n* (%)	Overall prevalence	Mild	Moderate	Severe
**Any urinary incontinence**	19,269/38,100 (50.6)	10,616 (56.5)	4512 (24)	3663 (19.5)
Stress	6060/37,622 (16.1)	4138 (68)	1264 (21)	658 (11)
Urge	6284/37,622 (16.7)	3207 (51)	1595 (25)	1482 (24)
Mixed	3767/37,622 (10.0)	1566 (41)	1085 (29)	1116 (30)
Other	2398/37,622 (6.4)	1542 (64)	492 (21)	364 (15)
**Any fecal incontinence**	2645/18,861 (14.0)	1609 (61)	532 (20)	504 (19)
**Any dual incontinence**	1980/18,866 (10.4)			

A total of 18,791 out of 19,269 women with urinary incontinence had available information regarding severity of UI

Of women with mild UI 156 had missing information regarding the subtype of urinary incontinence

Of women with moderate UI 76 had missing information regarding the subtype of urinary incontinence

Of women with severe UI 43 women had missing information regarding the subtype of urinary incontinence

**Table 3:** Demographic characteristics of post-menopausal women stratified by fecal incontinence (FI)

	No FI (*N* = 16,537)	FI (*N* = 2707)	Total cohort (*N* = 19,244)	*p* value
Age, median (IQR)	60.0 (55.0, 65.0)	60.0 (55.0, 66.0)	60.0 (55.0, 65.0)	0.01
BMI, median (IQR)	29.3 (25.8, 34.0)	30.6 (26.6, 35.6)	29.6 (25.9, 34.2)	< 0.01
Obesity, *n* (%)	7464 (46)	1457 (55)	8921 (47)	< 0.01
Education, *n* (%)				0.46
≤ 12 years	2981 (18)	494 (18)	3475 (18)	
13–15 years	5665 (34)	954 (35)	6619 (34)	
16+ years	7880 (48)	1255 (46)	9135 (48)	
Health insurance, *n* (%)	14,125 (86)	2303 (86)	16,428 (86)	0.63
Smoking, *n* (%)				< 0.01
Current	1660 (10)	350 (13)	2010 (10)	
Past	5292 (32)	954 (35)	6246 (32)	
Never	9565 (58)	1399 (52)	10,964 (57)	
Pack year current smokers, median (IQR)	11.0 (3.5, 22.0)	13.0 (3.5, 25.1)	11.5 (3.5, 22.0)	< 0.01
Hysterectomy, *n* (%)	4830 (29)	762 (28)	5592 (29)	0.26
T2DM, *n* (%)	3529 (21)	847 (31)	4376 (23)	< 0.01
Parity, *n* (%)				< 0.01
0	3278 (20)	485 (18)	3763 (20)	
1	3949 (24)	629 (23)	4578 (24)	
2	5005 (30)	797 (30)	5802 (30)	
3	2586 (16)	459 (17)	3045 (16)	
4+	1659 (10)	326 (12)	1985 (10)	
Depression, *n* (%)	3705 (22)	923 (34)	4628 (24)	< 0.01
Hours/week vigorous exercise, *n* (%)				< 0.01
None	7131 (47)	1366 (54)	8497 (48)	
< 1–4 h	6645 (44)	996 (39)	7641 (43)	
5–10+ hours	1374 (9)	187 (7)	1561 (9)	
HRT, *n* (%)	8958 (54)	1577 (58)	10,535 (55)	< 0.01
OC use in past 2 years, *n* (%)	3 (0)	0 (0)	3 (0)	0.48
Diuretic use, *n* (%)	8262 (50)	1563 (58)	9825 (51)	< 0.01
HTN, *n* (%)	10,389 (63)	1897 (70)	12,286 (64)	< 0.01
HLD, *n* (%)	9527 (58)	1706 (63)	11,233 (58)	< 0.01
History of DVT, *n* (%)	946 (6)	227 (8)	1173 (6)	< 0.01
History of stroke, *n* (%)	518 (3)	155 (6)	673 (3)	< 0.01

*BMI* Body Mass Index (calculated as weight in kilograms divided by height in meters squared), *Obesity* BMI greater than or equal to 30, *T2DM* type II diabetes mellitus, *HRT* hormone replacement therapy, *OC* oral contraceptive, *HTN* hypertension, *HLD* hyperlipidemia, *DVT* deep venous thrombosis

**Table 4:** Estimated number of US Black women with incontinence using 2020 US Census data and Black Women’s Health Study (BWHS) data (2011)

Incontinence	Age range	Prevalence of UI in the BWHS (95% CI)	Black women in the US 2020 Census	Number with incontinence (95% CI)
UI	35–44	0.42 (0.41–0.43)	2,806,108	1,777,743 (1,145,112–1,210,563)
	45–54	0.51 (0.50–0.51)	2,761,805	1,394,735 (1,370,871–1,418,593)
	55–64	0.52 (0.51–0.53)	2,651,749	1,381,138 (1,357,166–1,405,083)
	65–74	0.56 (0.55–0.58)	1,696,155	952,746 (928,756–976,609)
	75–84	0.61 (0.59–0.64)	797,396	468,344 (468,344–508,745)
	> = 85	0.63 (0.44–0.80)	350,752	222,143 (153,826–280,848)
	Total		11,063,965	5,617,232 (5,424,075–5,800,441)
	Prevalence estimate			0.508 (0.490–0.524)
FI	55–64	0.14 (0.13–0.15)	2,651,749	374,972 (356,591–393,963)
	65–74	0.15 (0.14–0.16)	1,696,155	247,195 (229,242–265,995)
	75–84	0.15 (0.13–0.17)	797,396	121,891 (105,826–139,359)
	> = 85	0.12 (0.02–0.30)	350,752	40,471 (8,579–105,766)
	Total		5,496,052	784,529 (700,238–905,056)
	Prevalence estimate			0.143 (0.127–0.165)
DI	55–64	0.11 (0.1–0.11)	2,651,749	280,392 (264,101–297,333)
	65–74	0.11 (0.1–0.12)	1,696,155	184,655 (168,677–201,595)
	75–84	0.13 (0.11–0.15)	797,396	103,192 (87,854–120,123)
	> = 85	0.12 (0.02–0.3)	350,752	40,471 (8,579–105,766)
	Total		5,496,052	608,710 (529,211–724,817)
	Prevalence estimate			0.111 (0.096–0.132)

*CI* confidence interval, *DI* dual incontinence, *FI* fecal incontinence, *UI* urinary incontinence

## Data Availability

Access to de-identified study data from the Black Women's Health Study for legitimate scientific research inquiry is available to qualified investigators with approval from appropriate Institutional Review Board. A formal concept proposal must be submitted by interested researchers, which is reviewed by the BWHS committee and data are made to researchers upon approval and completion of proper legal agreements including data use agreement and material transfer agreements. For more information on data requests please visit: https://www.bu.edu/bwhs/for-researchers/data-requests/.
